# Tumor stage and primary treatment of hepatocellular carcinoma at a large tertiary hospital in China: A real-world study

**DOI:** 10.18632/oncotarget.15433

**Published:** 2017-02-17

**Authors:** Jian-Hong Zhong, Ning-Fu Peng, Xue-Mei You, Liang Ma, Xiao Xiang, Yan-Yan Wang, Wen-Feng Gong, Fei-Xiang Wu, Bang-De Xiang, Le-Qun Li

**Affiliations:** ^1^ Department of Hepatobiliary Surgery, Affiliated Tumor Hospital of Guangxi Medical University, Nanning 530021, China; ^2^ Guangxi Liver Cancer Diagnosis and Treatment Engineering and Technology Research Center, Nanning 530021, China

**Keywords:** hepatic resection, hepatocellular carcinoma, transarterial chemoembolization, treatment selection, tumor stage

## Abstract

The current clinical reality of tumor stages and primary treatments of hepatocellular carcinoma (HCC) is poorly understood. This study reviewed the distribution of tumor stages and primary treatment modalities among a large population of patients with primary HCC. Medical records of patients treated between January 2003 and October 2013 for primary HCC at our tertiary hospital in China were retrospectively reviewed. A total of 6241 patients were analyzed. The distribution of Barcelona Clinic Liver Cancer (BCLC) stages was as follows: stage 0/A, 28.9%; stage B, 16.2%; stage C, 53.6%; stage D, 1.3%. The distribution of Hong Kong Liver Cancer (HKLC) stages was as follows: stage I, 8.4%; stage IIa, 1.5%; stage IIb, 29.0%; stage IIIa, 10.0%; stage IIIb, 33.6%; stage IVa, 3.4%; stage IVb, 2.5%; stage Va, 0.2%; stage Vb, 11.4%. The most frequent therapy was hepatic resection for patients with BCLC-0/A/B disease, and transarterial chemoembolization for patients with BCLC-C disease. Both these treatments were the most frequent for patients with HKLC I to IIIb disease, while systemic chemotherapy was the most frequent first-line therapy for patients with HKLC IVa or IVb disease. The most frequent treatment for patients with HKLC Va/Vb disease was traditional Chinese medicine. In conclusion, Prevalences of BCLC-B and -C disease, and of HKLC I to IIIb disease, were relatively high in our patient population. Hepatic resection and transarterial chemoembolization were frequent first-line therapies.

## INTRODUCTION

Hepatocellular carcinoma (HCC) is a common malignant tumor characterized by insidious onset, diverse etiology, and high mortality [1–2]. Most cases of HCC are associated with hepatitis B or C virus infection, alcohol-induced cirrhosis, or other chemical carcinogens [1–2]. HCC-associated morbidity continues to increase, and the disease accounts for nearly 600,000 deaths per year worldwide. Just over half of these deaths occur in China, where the disease is more prevalent in southeastern coastal areas [3–4]. One such area, Guangxi, has one of the highest rates of HCC-associated morbidity in the world (340 per 1,000,000 people) [5]. In China, the median age of HCC patients is 40–50 years, and more men than women are affected [5–7].

Hepatic resection and radiofrequency ablation are common, potentially quite effective primary radical therapies for early HCC. Unfortunately, most HCC patients do not show marked symptoms or signs early on, so they are diagnosed only after the disease has reached an advanced stage. For such disease, various treatment modalities may be used, such as systemic chemotherapy, radiotherapy, and traditional Chinese medicine (TCM).

A clear understanding of HCC stages at first diagnosis and most frequent primary treatments may help guide clinical practice when treating and managing this complicated disease. Therefore we retrospectively analyzed more than 6,200 patients with primary HCC treated at our large tertiary cancer hospital over a 10-year period.

## RESULTS

During the study period from January 2003 to October 2013, 8387 patients were initially diagnosed with HCC at our hospital, of whom 1832 were excluded because they had previously been treated for primary HCC at other hospitals (including hepatic resection, interventional therapy, local ablation therapy, chemotherapy), 58 because they were diagnosed or suspected of having intrahepatic cholangiocarcinoma, and 256 because their tumor stage was uncertain due to incomplete imaging data. In the end, 6241 patients were included in the analysis, of whom 5452 (87.4%) were male and 789 (12.6%) female. Median age was 48 years.

**Figure 1:**
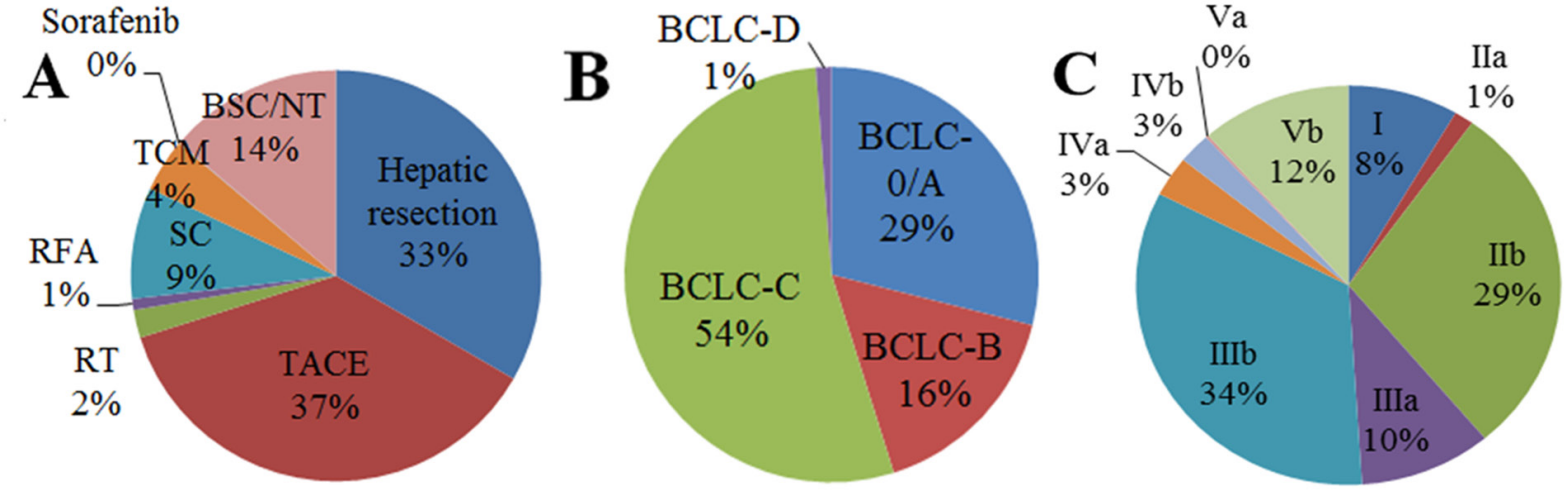
Frequencies of different primary treatments among patients with primary hepatocellular carcinoma A. without stratification or B-C. with stratification, based on the (B) Barcelona Clinic Liver Cancer system or (C) Hong Kong Liver Cancer system BSC, best supportive care; NT, no active treatment; RFA, radiofrequency ablation; RT, radiotherapy; SC, systemic chemotherapy; TACE, transarterial chemoembolization; TCM, traditional Chinese medicine.

Across all HCC, 33.3% of patients received hepatic resection and 36.7% received transarterial chemoembolization. Much lower proportions of patients received other primary therapies: systemic chemotherapy, 8.8%; TCM, 4.2%; radiotherapy, 2.2%; radiofrequency ablation, 0.9%; and sorafenib, 0.1%. A substantial proportion (13.8%) received best supportive care or did not receive any antitumor treatment (Figure 1A).

### Association between BCLC stage and initial therapeutic approach

**Figure 2:**
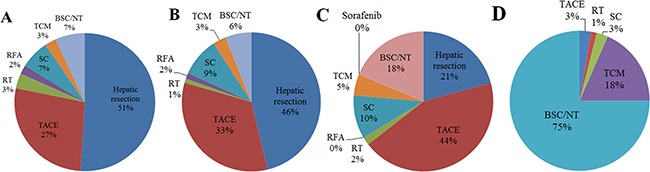
Distribution of primary treatments among patients with hepatocellular carcinoma in different stages of the Barcelona Clinic Liver Cancer (BCLC) system: (A) stage A (n = 1805), (B) stage B (n = 1012), (C) stage C (n = 3348), (D) stage D (n = 76) BSC, best supportive care; NT, no active treatment; RFA, radiofrequency ablation; RT, radiotherapy; SC, systemic chemotherapy; TCM, traditional Chinese medicine; TACE, transarterial chemoembolization.

Patients were classified into the following BCLC stages: stage 0/A, 28.9%; stage B, 16.2%; stage C, 53.6%; and stage D, 1.3% (Table 1, Figure 1B). Nearly half of patients with stage 0/A or B disease received hepatic resection, while approximately 30% received transarterial chemoembolization. Just under half of patients with stage C disease (43.8%) received transarterial chemoembolization, while only 20.7% received hepatic resection. Three-quarters of patients with stage D disease (75.0%) received best supportive care or did not receive any treatment for HCC (Figure 2A–2D).

**Table 1:** Barcelona Clinic Liver Cancer staging system [39]

Stage	Tumor features	Child-Pugh grade	ECOG performance status
BCLC-A	Single tumor or 2-3 tumors ≤3 cm	A or B	0
BCLC-B	2-3 tumors with a maximum diameter >3 cm or >3 tumors of any diameter	A or B	0
BCLC-C	Concomitant or isolated portal vein, hepatic vein, or vena cava tumor thrombus; bile duct tumor thrombi; preoperative tumor rupture; tumor metastasis to the lymph nodes; distant metastases	A or B	1-2
BCLC-D	Any	C	3-4

ECOG, Eastern Cooperative Oncology Group.

Most patients receiving hepatic resection and ablation therapy were in stage 0/A, while most patients receiving transarterial chemoembolization, systematic chemotherapy, TCM, targeted therapy, or no treatment were in stage C. Approximately half of patients receiving radiotherapy were in stage 0/A, while the other half were in stage C (Figure 2A–2D).

### Association between HKLC stage and initial therapeutic approach

Patients were classified into the following HKLC stages (Table 2): stage I, 8.4%; stage IIa, 1.5%; stage IIb, 29.0%; stage IIIa, 10.0%; stage IIIb, 33.6%; stage IVa, 3.4%; stage IVb, 2.5%; stage Va, 0.2%; stage Vb, 11.4% (Figure 1C). The most frequent primary treatments among patients with stage I to IIIb disease were transarterial chemoembolization (43%) and hepatic resection (40%). The most frequent treatment among patients with IVa or IVb disease was systemic chemotherapy (33.0%). Most patients with Va or Vb disease (73.4%) received best supportive care or did not receive any treatment, while 25.5% received TCM.

**Table 2:** Hong Kong Liver Cancer staging system [40]

Stage	Prognostic factor			
	ECOG PS	Child-Pugh grade	Tumor status	EVM
I	0	A	Early	No
IIa	1^a^	B^a^	Early	No
IIb	0-1	A	Intermediate	No
IIIa	0-1	B	Intermediate	No
IIIb	0-1	A/B	Locally advanced	No
IVa	0-1	A	Any	Yes
IVb	0-1	B	Any	Yes
Va	2-4^b^	C^b^	Early	No
Vb	2-4^b^	C^b^	Intermediate or locally advanced^c^	Yes^c^

ECOG PS, Eastern Cooperative Oncology Group performance status; EVM, extrahepatic vascular invasion/metastasis.

^a^ ECOG PS 1 and/or Child-Pugh B.

^b^ ECOG PS 2-4 and/or Child-Pugh C.

^c^ Intermediate/locally advanced tumor or EVM.

Most patients receiving hepatic resection or transarterial chemoembolization had stage I to IIIb disease. Most patients receiving systemic chemotherapy, radiotherapy, or targeted therapy had IIIb, IVa, or IVb disease.

## DISCUSSION

Studies suggest that the BCLC system can predict prognosis more accurately for Caucasian HCC patients than Asian ones, while the converse may be true of the HKLC system [8–11]. Though both staging systems are comprehensive, many patients do not fall neatly into their pre-specified treatment pathways [11], and even patients within the same BCLC or HKLC stage can differ substantially. These treatment gaps highlight the need to assess actual tumor stages and primary treatments of HCC in large patient populations.

This is one of the few large-scale descriptions of HCC stage and primary treatment to be conducted anywhere in the world, and the first to be conducted in Guangxi province of China, where 23,000 new cases of HCC are reported annually. A small percentage of these cases—in 2012, 897 (3.9%)—were initially diagnosed at our hospital. Consistent with current clinical opinion, just over one quarter of patients in our study population were diagnosed with BCLC-0/A disease, while just over half were diagnosed with BCLC-C disease. This suggests that a substantial number of HCC patients continue to be diagnosed too late for radical therapies (hepatic resection, liver transplantation, local ablation), leaving only the possibility of palliative therapies (transarterial chemoembolization, radiotherapy, systemic chemotherapy, molecular targeted therapy, immunotherapy, TCM), at least based on official recommendations.

One third of all patients in our study population underwent hepatic resection, including approximately 50% of patients with BCLC-0/A disease. Hepatic resection is used more often than the two other radical therapies, in part because of severe shortages of donated livers, and in part because the safety and efficacy of resection have improved substantially over the last 20 years with improvements in surgical techniques and perioperative nursing care [12–14].

One third of all patients in our study population received transarterial chemoembolization, including 30% of patients with BCLC-B disease and nearly half of patients with BCLC-C disease. Western guidelines for managing advanced HCC recommend transarterial chemoembolization or molecular targeted therapy [15–16], and a large systematic review [17] suggested that transarterial chemoembolization is significantly more effective than best supportive care. At the same time, several large studies have suggested that, for appropriately selected patients, hepatic resection is superior to transarterial chemoembolization or transarterial embolization for treating intermediate and advanced HCC [18–20]. This mounting evidence has yet to be recognized in official Western guidelines [21], though it has already been included in some Asian guidelines [1, 22].

After hepatic resection, the next most frequently used primary treatments for patients with BCLC-0/A disease were local ablative therapies. Radiofrequency ablation has grown in popularity and can provide efficacy comparable to that of hepatic resections in patients with single-tumor HCC [23]. In fact, this ablation procedure is recommended in National Comprehensive Cancer Network guidelines [21].

Primary treatments used much less often in our study population were radiotherapy, systemic chemotherapy and targeted therapy (primarily sorafenib). Radiotherapy and systemic chemotherapy have regained some popularity after a period of declining use in the 1990s [21, 24]. The targeted therapy sorafenib, although recommended by Western guidelines as a standard treatment for advanced HCC [15–16], remains controversial because of limited efficacy, frequent adverse reactions, and high treatment cost [25–26]. Immunotherapy is still being explored [27–28], and TCM-based treatments of HCC appear to be even less promising [29–30].

A substantial proportion of our study population (13.8%) did not receive treatment for HCC because they were unable to cover the costs, they decided to abandon treatment as a result of terminal disease, or they went to other hospitals for treatment. For these same reasons, most patients with BCLC-D disease did not receive any treatment.

The distribution of our patients across BCLC stages shows interesting differences from the distribution in a study of 3892 patients treated between 1986 and 2002 for primary HCC at a single large hospital in Taiwan [31]. The proportion of patients with BCLC-B disease was much higher in that study than in ours. This may reflect the fact that they staged a single tumor of >5 cm diameter as BCLC-B, whereas we staged such disease as BCLC-A. Indeed, the combined proportion of patients with BCLC-0, -A or -B disease was much higher in that study (63%) than in ours (45%). Conversely, the proportion of patients with BCLC-C disease was much lower in that study, which may suggest that HCC is diagnosed earlier more often in Taiwan than in mainland China.

Frequencies of different primary treatments in our study also show interesting differences from those of the Taiwan study. While similar proportions of patients received transarterial chemoembolization in their study (40%) as in ours (36.8%), only 12% of their patients underwent hepatic resection, compared to 33.4% of our patients. Nevertheless, both our study and the one from Taiwan suggest that hepatic resection is superior to transarterial chemoembolization [18, 31–32].

The findings of our study should be interpreted with caution, given that they are based on patients at a single medical center, albeit a large one that attracts patients from the entire province (Guangxi) of 46 million people. Indeed, the status of our hospital as a regional specialist oncology center increases the risk that the high proportion of our patients with advanced HCC reflects the fact that many patients with early disease received radical therapy in local hospitals, while many with advanced disease were referred to our hospital because of inadequate local medical services. Moreover, liver transplatation and transarterial radioembolization were not used in our hospital. The experience in our center may be hardly representative of the expereience in other centers in the choice of first line treatment [33–34]. Another limitation is the lack of information about patients’ prognoses. Prognoses of most of the included patients who underwent hepatic resection or transarterial chemoembolization were described in our previous studies [13, 18, 32, 35–36]. And third, HCC patients may have different clinical and pathological characteristics and patient outcomes for different stages of HCC according to the BCLC classification system [37]. However, we can not perform such analyses because of the lack of relevant data.

Despite these limitations, our study provides insights into the epidemiology of HCC staging and treatment practices in an extremely high-incidence region. This may help guide treatment and management of the disease.

## MATERIALS AND METHODS

### Subjects

This study involved retrospective review of medical records of patients initially diagnosed with HCC at the Affiliated Tumor Hospital of Guangxi Medical University (Nanning, Guangxi, China) from January 2003 to October 2013 [35]. HCC was clinically diagnosed in accordance with criteria of the European Association for the Study of the Liver [15]. Only patients who received initial treatment for HCC at our hospital were included in the analysis.

### Patient assessment and data collection

Patient data, taken from the database of our hospital's Disease Management Office, included patient gender, age, time of admission, clinical diagnosis (including pathology diagnosis in the case of patients who underwent hepatectomy), tumor size and number, macrovascular or bile duct invasion, portal lymph node/extrahepatic metastasis, and preoperative tumor rupture. The presence of invasion, metastasis or tumor rupture was assessed using B ultrasonography, computed tomography and/or magnetic resonance imaging; or it was determined based on perioperative conditions. Hepatic function and performance status were assessed on the basis of physical examination and biochemical tests after admission. Patients were excluded if data were inadequate to allow definitive tumor staging.

### Staging systems

Several systems have been proposed for staging HCC, including the Japan Integrated Staging (JIS) score [38], BCLC staging [39], Hong Kong Liver Cancer (HKLC) system [40], Italian Liver Cancer (ITA.LI.CA) system [41], and the Model to Estimate Survival for HCC patients (MESH) score [42]. Only the BCLC [39] and HKLC [40] systems go beyond staging to recommend stage-appropriate treatment modalities. Therefore, these systems were used for staging in the present study.

### Statistical analysis

Data were analyzed using Microsoft Excel (Windows 2000).
